# Oral Vaccination of Fish – Antigen Preparations, Uptake, and Immune Induction

**DOI:** 10.3389/fimmu.2015.00519

**Published:** 2015-10-19

**Authors:** Stephen Mutoloki, Hetron Mweemba Munang’andu, Øystein Evensen

**Affiliations:** ^1^Department of Basic Sciences and Aquatic Medicine, Faculty of Veterinary Medicine and Biosciences, Norwegian University of Life Sciences, Oslo, Norway

**Keywords:** oral vaccination, fishes, oral tolerance, antigen production, local and systemic immune responses

## Abstract

The oral route offers the most attractive approach of immunization of fish for a number of reasons: the ease of administration of antigens, it is less stressful than parenteral delivery and in principle, it is applicable to small and large sized fish; it also provides a procedure for oral boosting during grow-out periods in cages or ponds. There are, however, not many commercial vaccines available at the moment due to lack of efficacy and challenges associated with production of large quantities of antigens. These are required to stimulate an effective immune response locally and systemically, and need to be protected against degradation before they reach the sites where immune induction occurs. The hostile stomach environment is believed to be particularly important with regard to degradation of antigens in certain species. There is also a poor understanding about the requirements for proper immune induction following oral administration on one side, and the potential for induction of tolerance on the other. To what extent primary immunization via the oral route will elicit both local and systemic responses is not understood in detail. Furthermore, to what extent parenteral delivery will protect mucosal/gut surfaces and vice-versa is also not fully understood. We review the work that has been done on the subject and discuss it in light of recent advances that include mass production of antigens, including the use of plant systems. Different encapsulation techniques that have been developed in the quest to protect antigens against digestive degradation, as well as to target them for appropriate immune induction are also highlighted.

## Background

Mucosal surfaces constitute the largest body area of living organisms in constant contact with the external environment and are responsible for the maintenance of immunological homeostasis. The gut is the most attractive route for antigen delivery in fish for several reasons. It offers an easy way of administering antigens; delivery is less stressful, both small and large sized fish can be vaccinated and this approach is associated with no side effects. Despite these advantages, there are currently very few oral vaccines registered for use in the aquaculture industry. For example, out of 17 commercially available vaccines against viruses reported in 2014, only 2 were oral preparations (1). This low number was attributed to poor performance of oral vaccines in general compared to their injection counterparts (2), possibly as a result of antigen degradation during passage through the hostile stomach environment prior to reaching the second segment of the intestine where absorption takes place. Despite this, the search for oral vaccines that meet the desired induction of the immune response and protection has continued. The struggle in developing good oral vaccines is complicated by the lack of proper understanding of what constitutes good immunological induction leading to protection on one side, and the induction of tolerance on the other.

For the most part, efforts to develop vaccines whether for parenteral or oral administration have been largely directed at bacterial and viral infections. Recently, however, other challenges facing the aquaculture industry, namely, ectoparasites have prompted efforts to address in particular sea lice and amoeba infections in salmonids.

## Antigen Uptake and Distribution

The gut of teleosts can be subdivided into two groups in terms of the uptake of macromolecules: (1) those devoid of stomachs, i.e., pre-larval stages of fish as well as certain species that do not develop stomachs throughout their life time, e.g., cyprinids; (2) fish with stomachs and therefore possess a segment of low pH and are capable of pre-digestion (3). The gut can further be divided into three segments based on the morphology of enterocytes namely segment 1: 60–75% of the total gut length depending on species with cells considered absorptive; segment 2: 10–15% of the gut length and with enterocytes characterized by large supranuclear vacuoles and high pinocytotic activity; and segment 3: less well-characterized but with enterocytes that have osmoregulatory function and short microvilli [summarized in Ref. (4, 5)].

The most uptake of macromolecules in the gut of fish takes place in the second segment although in a few species like the cod, this segment has not been identified (6). In general, particulate and soluble antigens are taken up by different mechanisms: particles such as horse radish peroxidase (HRP) in carp are thought to be taken up by receptor-mediated endocytosis (7). HRP was observed in the endolysosomal compartment followed by intercellular spaces where after it was observed systemically (8). Ferritin (soluble), on the other hand, was taken up into supranuclear vacuoles (endosomes), a route believed to be used also by other soluble factors such as bacterial LPS (9).

Antigens administered via the gut are invariably taken up by cells lining this organ such as enterocytes and M cells in the case of higher vertebrates. In fish, enterocytes have been shown to take up antigens (10, 11). M-like cells, on the other hand, have been shown to have functional endolysosomal organelles and not able to phagocytoze inactivated bacteria in fish (5). It is nevertheless thought that if aided with certain signals like poly D, l-lactide-co-glycolic acid (PLGA) microparticles, M-like cells may still be able to take up antigens (12). It is generally considered that the best immune induction will be that which mimics a natural infection. Different pathogens are internalized by different routes/receptors under natural infections and it is not unlikely that some of the pathogen for which oral vaccines are sought will not reach or target the enterocytes. To what extent this will be an issue in the efficacy of oral vaccines remains to be shown. This is especially relevant given the compartmentalization of immune responses as discussed below. As fish reside in the aquatic environment, it is likely that most pathogens will invade via mucosal routes, including the gills, skin (fin bases) (13), and also via the gut (14–16).

## Immune Induction Following Oral Vaccination

It is well-known that lymphoid structures associated with the gut of fish are different from those of mammals. Fish do not have lymph nodes or Peyer’s patches but instead possess a less organized, diffuse gut-associated lymphoid tissue (GALT), which is functionally different from that of mammals (17). Nevertheless, the fish GALT is capable of local immune responses. Indeed, oral administration of antigens result in the up-regulation of genes related to recruitment of immune cells (18) and local antibody production (19, 20). Protection is, however, variable with some reporting adequate (3, 21–23) and others inadequate (24). As primary vaccines, orally delivered vaccines especially containing inactivated whole antigens have traditionally not featured well, often resulting in suboptimal protection against several pathogens. When used for boosting, however, oral vaccines have been shown to be capable of enhancing or extending protection (25) although the antibody response is transient, typically lasting about 3 months (3, 9, 12, 26). Interestingly, varying the dosage regime, for example, by administering the vaccine 3 days/week for 2 months instead of 5 days/month gave different results with the former being more effective (24). The basis for this difference has not been examined but may reflect a delicate balance that has to be maintained in augmenting the immune response by oral boosting and should be subject of further studies.

Other factors that influence efficacy of oral vaccines include the nature of antigens, formulation, and dosage. Bacterial whole antigens generally perform better than viral antigens. Formulation is important for some vaccines in general and for oral vaccines, it is even more so given that they act as a means to protect antigens through the stomach as further discussed below.

Dosage is key to any vaccine regime and for orally administered vaccines, it is difficult to determine the dose at individual level. Examination of feed residues and weight gain at population level following feeding gives an idea of the average in-take in the population. Then comes the distribution of antigens per weight unit of feed or per pellet, which is also difficult to determine. This is a topic addressed to a very little extent in published studies and potentially has a great impact on the outcome of mucosal immunization modalities.

**Figure 1:**
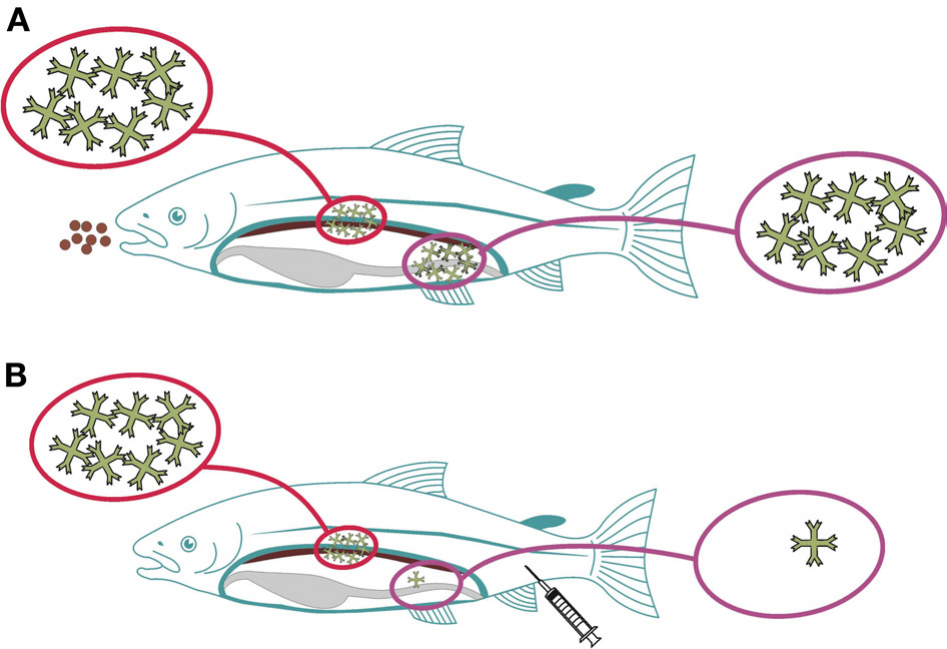
**Proposed asymmetry for immune responses induced via mucosal (gut) versus parenteral routes in fish**. When antigens are delivered via the gut, local and systemic immune responses will be elicited, symbolized by high amounts of circulating IgM **(A)**. When the antigens are delivered parenterally, systemic responses will be strong, while local (gut) responses will be almost absent **(B)**.

Compartmentalization of the immune system is yet another factor to consider, a well-known phenomenon in mammals. The basis for this is the trafficking of immune competent cells aided by homing receptors (27, 28). Further to this, whether an asymmetric immune response is induced in fish as seen in mice (29) is not known. If so, the concept would be that immune responses induced in the gut would induce local and systemic immune responses, while parenteral delivery will not yield protective immune responses on mucosal surfaces (Figure 1). In a recent study, it was shown that rainbow trout infected with a gut parasite (*Ceratomyxa shasta*) had local (gut) IgT responses but not IgM responses, while systemically, IgM levels were high but IgT was not detected in circulation (30). Reciprocal modalities were not tested but in a recent study it was shown that parenteral vaccination efficiently stimulates systemic responses but is a poor inducer of mucosal immunity (12, 31). Furthermore, oral administration of antigens results in stimulation of both systemic and mucosal responses (32, 33). Together, these findings align with a hypothesis also for fish that the immune response shows an asymmetrical pattern but further studies are needed to better understand the importance and implications.

Mucosal immunoglobulins (such as IgT or IgZ) are relative new discoveries in fish immunology. For this reason, their functional role is not well-understood/studied and in general, very few studies (perhaps none) show that IgT plays a role in protecting mucosal surfaces. Although Zhang and co-workers (30) provides some guidance, it does not show that IgT is protective. It could be that IgT merely plays a role in regulating the commensal flora thus keeping it in check, preventing the proliferation of any single bacterial species beyond a level that could potentially result in pathology and disease, in line with current thinking in mammalian immunology (34). There are a few studies on the potential importance of IgT in the gills (intraepithelial location) against *I. multifilis* infection but the functional importance of IgT+ B cells is actually not known (35).

## Oral Tolerance

Oral tolerance is defined as the hypo-responsiveness to a fed antigen (36) and is a result of the suppression of the cellular and/or humoral immune response (37). It is a phenomenon that has been well-known for ages and in fish, it has been recognized as the suppression of antibodies (12, 24, 26) and is easily induced. In higher vertebrates, the causes of tolerance are multiple including low doses that favor the induction of Tregs; and higher doses associated with anergy (36). Repeated administration of small amounts of antigens, vaccination of too young (immunocompetent) fish, low temperatures (lower end of the permissive limit), type of antigens and administration regime as well as genetics are factors that have been implicated in the induction of tolerance in fish (11, 18, 24, 38).

**Figure 2:**
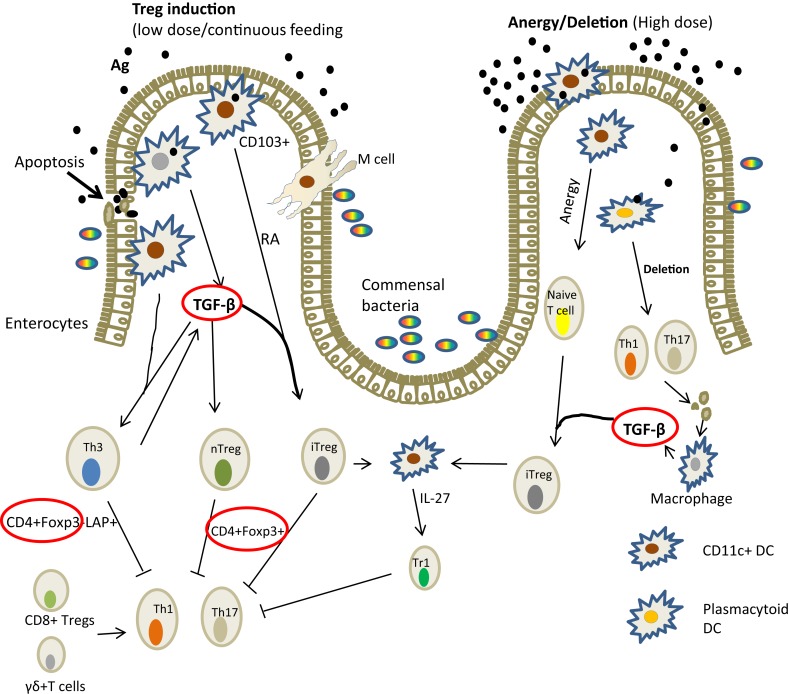
**Mechanism of induction of oral tolerance in the gut in mammals [adapted from Ref. (36)]**. There are several ways in which oral antigens may be taken up through the gut epithelium: by the enterocytes; sampled by DCs (macrophage-like cells in fish) that penetrate the lumen or via M cells. The gut environment favors tolerance probably to allow for gut microflora. DCs are known to drive Treg differentiation from FoxP3, TGF-β, and IL-10 from gut epithelial cells. Lower antigen doses tend to induce TGF-β while high doses lead to anergy. In Atlantic salmon, the expression of IL-10, TGF-β, Foxp3 (circled red) associated with suppressed antibody responses have been demonstrated (32). Key: TGF, transforming growth factor; RA, retinoic acid; DC, dendritic cells; LAP, latency associated peptide; Foxp3, forkhead box protein; IL, interleukin.

Mechanisms of tolerance involve the induction of Tregs associated with up-regulation of FoxP3 and production of TGF-β (Figure 2). Other cells are also involved and these include dendritic cells, macrophages, and epithelial cells. In fish, the mechanisms have not been elucidated in any detail; it is for the most part illustrated by decreased antibody response following repeated antigen exposure (24, 26, 32). It is only recently that it has been shown that the suppression of antibody production was accompanied by the induction of FoxP3, TGF-β, and IL-10 (32), strengthening the belief that the mechanism is comparable to that of higher vertebrates.

## Recent Developments to Improve the Performance of Oral Vaccines in Fish

The disadvantages associated with oral vaccines include their demand for high antigens requirements, the need to protect antigens as they pass through the stomach as well as the formulation of vaccines to improve the stimulation of protective immunity. The following are the strides that have been taken by the scientific community to bring the frontier of oral vaccinology in fish forward.

## Antigen Production

A prerequisite to the production of any vaccine is the ability to scale-up antigens easily and at a low cost. While bacteria and bacteria-based products, such as subunit antigens, are quite easily propagated by fermentation, scaling up for virus antigens can be challenging and thus this section focusses mainly on virus antigens.

### Virus Antigen Preparation

Virus antigens used in the production of vaccines in general and oral vaccines in particular range from native whole pathogens (whole virus preparations) to subunit or synthetic products.

### Native Whole Pathogen Antigens

At present, most commercial vaccines against viral diseases for parenteral delivery in fish are produced by inactivating whole pathogens. For fish vaccines, viral antigens are typically produced by propagation in cell culture and this is limited by the yield obtained. As an example, infectious pancreatic necrosis virus (IPNV) produces yields ranging between 10^6^ and 10^10^TCID_50_ml^−1^ if grown in RTG 2, CHSE, or other permissible cell lines (39–41). This is complicated by the fact that yields may be inconsistent and differ greatly between workers and laboratories. To resolve the problems stated above, several solutions have been attempted in recent years, including (1) discovery of cell lines with short regeneration time and high antigen yields; and (2) development of cell culture systems that can house higher cell numbers per unit volume. Using IPNV as an example, Asian Grouper strain K cells (AGK cells) have been produced from the skin of Orange spotted grouper (*Epinephelus coioides*) that yield high amounts of virus (approximately 10^10^TCID_50_ml^−1^) (42) while having a short cell-generation turn-around time, i.e., frequent splits and high split ratios (43).

For cell culture systems, flasks that can accommodate more cells per unit volume than ordinary flasks have recently been developed by commercial companies. An example is the multilayered hyper flasks from Corning^®^ with scaled-up volume (up to 10 times) on which cells can be grown (www.sigmaaldrich.com). Another example is the BelloCell^®^ from CESCO Bioengineering Inc. (44). The principle of the BelloCell is that of alternating nutrient and gaseous exchange. This results in maximum cell growth and increased cell population. Indeed, improved cell growth has been reported using this method and it has potential for use in fish vaccination, although currently there are no reports that suggest that it has yet been used so far. Other developments include the three dimensional cell culture systems, such as Hydromatrix™, MaxGel™, and Mouse ECM from Sigma^®^ that mimic *in vivo* growth conditions of cells. As with BelloCells, the systems result in better growth of cells and will likely lead to increased antigen yields. Finally, the contribution of other improvements in the cell culture cultivation process, for example, refinement in the culture media production and process control cannot be underestimated.

### Subunit Antigens Produced in Bacteria and Yeast Cell Systems

Subunit antigens are produced from heterologous protein expression systems and offer the safest and most attractive means of antigen production. Unlike live pathogens or DNA vaccines, subunit vaccines do not pose the risk of invading the host or integrating with the host DNA. In fish vaccinology, the most widely used protein expression systems for the production of subunit antigens are *E. coli* and yeast. Commercial oral vaccines have been used previously or are in production on the basis of these techniques (45–47). Nevertheless, other systems for example the Baculovirus expression system in eukaryotic cells has also been used especially at experimental level (33).

### Subunit Antigens in Plant-Based Systems

During the last decade, the use of whole plants as antigen production systems has received a lot of attention owing to the advantages that they offer, such as ease of scaling up, reduced costs, and good safety margins (48). These systems have also been referred to as *molecular* farming and are the utilization of whole plants or plant cells/tissues cultured *in vitro* for the production of recombinant proteins. Plants have advantages compared to traditional platforms of recombinant protein production in the sense that they are less expensive to establish and maintain, they lack undesirable components such as hyper glycosylated proteins as found in yeast, and extraneous agents are less of a problem. Scalability is relatively easy. Finally, plants are higher eukaryotes and can fold and assemble complex/multimeric proteins and also perform post-translational modifications (48). Despite these many advantages, there are currently no recombinant proteins produced commercially. Many proof-of-principle studies have been done and several companies are investigating and exploring the commercial feasibility of such production systems. The focus of such efforts has been directed at a small number of well-characterized plants of which Tobacco cultivars Bright Yellow (BY-2) and *Nicotiana tabacum* 1 (NT-1) are most popular (49). It is noteworthy, however, that plant systems lack intrinsic benefits of cultured cells, for example, the difficulty associated with the control of growth conditions and batch to batch inconsistency (49). Regardless, this is likely to be the trend for the future also for the aquaculture research community (48).

### DNA-Based Antigens

DNA-based vaccines in the aquaculture industry have met some success with one injectable vaccine against infectious hematopoietic necrosis in Atlantic salmon being licensed for commercial use in Canada (50). Although there is no commercial vaccine yet licensed for use as an oral vaccine, induction of protection against IPNV has been reported using this method at experimental level (21). The biggest problem with DNA vaccines in the aquaculture industry at the moment is the licensing requirements related to integration into genome studies, in principle related to safety to the end-consumer more than the vaccinated animal (51, 52).

### Bacterial Antigens

In general, there has been more success with bacterial vaccines of the aquatic industry compared to their viral counterparts, for example, if we look at the relative number of commercial vaccines for salmonids in Norway (53). There are several reason for this, while all viruses are intracellular pathogens, most of the bacteria important to the fish industry are extracellular, with only a few exceptions for example *Piscirickettsia salmonis* whose vaccine development has equally been a challenge (54). The effective immunological response against extracellular bacterial pathogens is predominantly humoral, thus vaccines that act by inducing antibody responses will normally suffice to protect the animals against challenge. By contrast, protection against intracellular pathogens requires a combination of immunological responses, including humoral, cell-mediated, and cytotoxic responses. To produce a vaccine which induces all these responses is what makes it a challenge. By contrast, factors that contribute to the relative ease in development of bacterial vaccines compared to viral vaccines are their relatively large size and the type of antigens/immunogens they possess.

Oral bacterial vaccines have similar challenges as viral vaccines, for example, degradation of antigens as they pass through the stomach and requirements for large volumes. A study by Villumsen and others showed that protection of fish was conferred if the vaccine was administered anally compared to the oral route, with the authors suggesting that orally fed antigens were digested in the stomach (55). Indeed, commercial bacterial vaccines for oral administration, e.g., enteric red mouth vaccine (Aquavac ERM Oral vet from MSD animal health) are only intended for use as booster vaccines (56). Consequently, several studies aimed at finding the best solution to protect bacterial antigens in the stomach of fish have been conducted (57) and these are discussed in the sections below.

Some bacteria, specifically *Lactobacillus* expressing various proteins of IPNV have been applied in studies of oral vaccination of fish (58, 59). Although the results are contrasting, this is nevertheless an interesting concept that warrants further follow-up, as does the delivery of a combination of bacteria and viruses as multivalent oral vaccines. This may help synergize responses to viral antigens as has been observed for injectable vaccines.

## Encapsulation Techniques

Encapsulation refers to incorporation of materials, including food ingredients, cells, or others, into small capsules and is accomplished by several different techniques (60). Encapsulated materials, e.g., vaccine antigens can then be mixed with food for oral administration. In vaccine development for fish, there are essentially three methods by which this is done: (1) finished feed is top-dressed with vaccine powder by using adhesive agents, e.g., edible oil or gelatin; (2) finished feed is sprayed with the vaccine if the latter is in liquid form; and (3) mixing the antigen with the feed in the production process (61). The first two methods (top dressing) are quite simple to apply but have the disadvantage of uneven distribution in the feed and also the threat that the antigens are directly exposed to hostile stomach environment upon feeding, leading to degradation. By contrast, mixing the antigen with the feed gives the advantage of uniform distribution of the antigens in the feed. Since most fish feed is produced through an extrusion process at high temperature and pressure antigens would have to be added to the pellet at later stages, either in a vacuum infusion coating process. As a means of protecting the antigens against the hostile stomach environment, several encapsulations techniques have been developed and tried as discussed below.

### Microalgae

Microalgae are potentially future candidates of recombinant vaccine production (62) both for higher vertebrates and the fish industry. This system is said to have among other advantages the ease of scalability, rapid transformation, and consistent transgene expression levels (62). In oral vaccination of fish, however, there are no reports on the use of this technology.

### Alginate Particles

Alginates are another carrier that promises to revolutionize oral vaccine development. They occur naturally in brown algae and have been targets for trapping macromolecules and cells (63). In the aquaculture industry, they have been tested quite extensively (Table 1).

**Table 1:** **Summary of previous studies of oral vaccination of fish using alginate microparticles**.

Target	Fish species	Result	Reference
Commercial *Vibrio anguillarum* vaccine in two types of alginate microspheres	Carp and rainbow trout	Antibodies produced by carp only; protection not assessed	(38)
Plasmid DNA expressing major capsid protein of lymphocystis disease virus	Japanese flounder	Antigens detected in various tissues between 10 and 90 days post immunization	(64)
*Aeromonas salmonicida* recombinant A layer proteins in alginate beads	Goldfish	Antibodies produced but no difference in disease susceptibility between the control and treatment groups after challenge	(24)
*Lactococcus garvieae* bacterin	Rainbow trout	RPS of 50 achieved	(65)
*L. garvieae* bacterin	Rainbow trout	Achieved RPS of 53 after 30 days following challenge; after a boost at 61 days, RPS increased to 61 at 120 days	Altun et al. (66)
*Flavobacterium columnare* bacterium in alginate microparticles; compared oral and parenteral deliveries	Nile tilapia	No protection or significant antibody production in oral group	(67)
Infectious pancreatic necrosis virus VP2 plasmid DNA in alginate microspheres; administered by intubation	Brown trout and rainbow trout	RPS of 84 (brown trout) and between 67 and 83 (rainbow trout) after 30 days	(68)
IPNV VP2 plasmid DNA in feed pellets	Rainbow trout	Induction of transcriptional responses; RPS of 78–85	(25, 69, 70)
Inactivated IPNV virus encapsulated in alginate beads; feed pellets	Atlantic salmon	Induced antibody response; no protection measured	(32)

As can be seen from the table above, alginates seem to work well with DNA plasmids, giving RPS values of 67% or more in all species tested. Whether this technology can be transferred to other viral diseases of fish would be interesting.

### Nanoparticles

In recent years, increased knowledge has accumulated over the use of different forms of nanoparticles in oral vaccination of fish. Rajesh et al. (71) examined the use of chitosan nanoparticles for orally delivering a DNA vaccine against *Vibrio anguillarum* in sea bass. While they demonstrated that fish took up the antigens, fish were, however, not protected and a relative RPS rate of 46% was recorded. A better protection against *V. parahaemolyticus* was recorded in black seabream (*Acanthropagus schelegelii* Bleeker) also vaccinated with a DNA vaccine loaded in nanoparticles, resulting in 72.3% RPS 3 weeks post vaccination.

For viral diseases, encapsulating DNA vaccines against infectious hematopoietic necrosis virus with Poly (d,l-lactic-co-glycolic acid) (PLGA) nanoparticles and adding to feed pellets showed that rainbow trout took-up the vaccine in the lower intestine within 96 h of feeding and also induced low levels of gene expression and specific antibodies but this was not sufficient to protect the fish against lethal challenge (72). When a DNA vaccine against lymphocystis loaded in PLGA was used to feed Japanese flounder, several innate immune parameters were induced suggesting that the system could be used as a carrier for plasmid DNA vaccines (73). Recently, Rivas-Aravena and co-workers (74) reported enhanced protection of Atlantic salmon fed with chitosan nanoparticles-based oral vaccines loaded with a DNA coding an alphavirus replicase (as an adjuvant) while the target antigen was ISAV. The authors reported 77% protection.

### Biofilms

Biofilms are defined as communities of microbes adherent on a surface and usually held together by a polymetric extracellular matrix (75, 76). In so doing, they form a protective coat which has been taken advantage of in vaccine studies of fish to prevent the degradation of antigens through the stomach. Work in fish vaccination using biofilms is not as extensive as literature dates back to 2000–2004 and was centered on *A. hydrophila* (2, 77). In general, all the studies reported significant protection of fish fed with biofilms compared to free cells. No additional work has been reported thereafter.

### Artemia

First described in the mid 1700s by Schlosser, Artemia have gained value as food for fish as well as for other uses (78). Artemia naturally consume the bacteria that they are immersed with thereby readily encapsulating them and it is this property that is exploited in vaccine development. This approach has been examined in oral vaccination of fish targeting different bacteria not only *V. anguillarum* (38) but also recombinant *E. coli*. (46). Where the immune response was evaluated, there were indications of immunosuppression following very early vaccination in carp while an increased antibody response was reported for older carp immunized at 58 days. Similarly, older sea bream developed highly increased antibodies 21 days following a boost (38).

### Oral Vaccines Against Sea Lice and Amoeba

As fish farming expands, so does the number of important ectoparasites (79). In the salmonid industry at present, the most important ectoparasites include sea lice (*Lepeophtheirus salmonis* and *Caligus rogercrossey*) and amoeba (*Neoparamoeba perurans*) the causative agents of amoebic gill disease. Conventional treatments against the former have mainly involved the use of chemotherapy with products, such as organophosphates (80) and avermectins (81) but as with many other drugs, resistance is an increasing problem (82, 83). Amoebic gill disease is more problematic to treat but repeated freshwater baths (84) and also hydrogen peroxide (85) are used.

When it comes to vaccination, to the knowledge of the authors, there are no reports that address oral vaccination against sea lice (*L. salmonis*) in Atlantic salmon, or (*C. rogercrossey*) in Coho salmon. Since both parasites attach to and complete their life cycle on the skin of salmon, it is assumed that local responses would be important in preventing infections but so far no attempts have been made to induce immune responses via the mucosa.

Similarly, there are apparently no records on testing oral vaccines against amoeba in fish. Interestingly, in rodents, *Yersinia enterocolitica* expressing an amoeboid outer protein as a fusion protein has been shown to induce some degree of protection (86, 87). This could be an avenue to explore also for fish as an antigen delivery model against amoeba.

### Summary of Status and Future Directions

There is a consensus among scientists working in this field that antigen uptake in teleosts takes place in the second gut segment. In order for some antigens to reach this site, protection against degradation in the stomach is necessary. The second gut segment is possibly the site where immune induction is initiated locally for antigens taken up from the intestines. However, the cell types, molecular elements, and even immune organs involved are yet to be definitively elucidated. Central to this is the role of IgT or IgZ, for example, do they neutralize antigens or merely regulate the relative number of bacteria in the gut microbiome? Could it be that bacteria coated with IgT represent proliferating, potentially disease-causing bacteria? Furthermore, what roles do IgT or IgZ have vis-à-vis IgM in protective immunity? What functional significance does compartmentalization of the different isotypes have? Thus, the mechanism of immune induction (local and systemic) and protection following oral vaccination is yet to be elucidated. Reagents that allow functional studies of IgT are in very few hands at the moment, and this has partly caused the slow pace in understanding its role in the protection of fish against pathogens. Further, it will be important to understand if antigens delivered at mucosal surfaces (oral, skin, gills, and/or nasal) will elicit both local and systemic responses. Studies here are not conclusive. While IHN virus delivered nasally elicit systemic immune responses (88) it has also been shown that live and inactivated IPN virus are taken up when delivered orally and anally (10). IPNV delivered orally give a boost response to circulating IgM (10). Despite these findings, there is a need to better understand if inactivated and live (replicating) antigens differ in their ability to induce systemic, protective responses when antigens are delivered locally. The potential of differences between pathogens also need to be explored.

When it comes to vaccine formulation, several candidates that can serve as vehicles for antigens, for example, alginates have been identified and shown to be capable of protecting antigens against degradation in the stomach. The contribution of these vehicles toward augmentation of the immune response, however, remains poorly understood. Similarly, the effects of adjuvants in this field have not been well explored, except for a few studies, e.g., recombinant TNFa (89). This is an area that is likely to take focus, as shall the continued exploration of more effective encapsulation techniques.

In terms of antigen preparations, a number of products are commercially available on the market that makes it easier and cheaper to produce larger volumes of antigens, especially those of virus nature. Here, the use of plants gives an interesting impetus and need following up.

Finally, when it comes to DNA vaccines, the future is not easy to predict. There is no doubt that progress will continue for injectable vaccines especially where other approaches have little or no efficacy. For oral vaccines as well, DNA preparations will come. However, acceptability in some regions such as Europe will depend on changes in legislation (90).

## Conclusion

The last decade has seen an increase in the number of studies addressing oral vaccination of fish. The discovery of new methods of efficiently producing antigens particularly of viral antigens and the concept of using plant systems for the production of recombinant antigen presents new exciting future possibilities. Unraveling the immune response to antigens in the oral compartment and systemically vis-à-vis oral tolerance remains a challenge and requires more efforts. Finally, there is need to understand better a potential asymmetry in the immune responses of fish elicited by antigens delivered via the gut (or mucosal surfaces in general) versus parenteral delivery of antigens, and the importance of this for protecting the primary barriers of infection.

## Conflict of Interest Statement

The authors declare that the research was conducted in the absence of any commercial or financial relationships that could be construed as a potential conflict of interest.
